# Late Antiretroviral Therapy (ART) Initiation Is Associated with Long-Term Persistence of Systemic Inflammation and Metabolic Abnormalities

**DOI:** 10.1371/journal.pone.0144317

**Published:** 2015-12-04

**Authors:** Mathilde Ghislain, Jean-Philippe Bastard, Laurence Meyer, Jacqueline Capeau, Soraya Fellahi, Laurence Gérard, Thierry May, Anne Simon, Corinne Vigouroux, Cécile Goujard, ANRS-COPANA Cohort Study Group

**Affiliations:** 1 Inserm UMRS1018, CESP, Epidemiology of HIV and STI, Le Kremlin-Bicêtre, France; 2 Tenon Hospital, AP-HP, Department of Biochemistry and Hormonology, Paris, France; 3 Inserm UMRS 938, Centre de Recherche Saint-Antoine, Paris, France; 4 Sorbonne Universities, UPMC, Institute of Cardiometabolism and Nutrition (ICAN), Paris, France; 5 Paris-Sud university, Le Kremlin-Bicêtre, France; 6 Bicêtre Hospital, AP-HP, Department of Public Health, Le Kremlin-Bicêtre, France; 7 Saint-Louis Hospital, AP-HP, Department of Clinic Immunopathology, Paris, France; 8 Teaching hospital of Nancy, Brabois Hospitals, Department of Infectious and Tropical Diseases, Vandoeuvre les Nancy, France; 9 Pitié-Salpétrière Hospital, AP-HP, Department of Internal Medicine and Clinical Immunology, Paris, France; 10 Saint-Antoine Hospital, AP-HP, Common Laboratory of Biology and Molecular Genetics, Paris, France; 11 Bicêtre Hospital, AP-HP, Department of Internal Medicine, Le Kremlin-Bicêtre, France; 12 Inserm-ANRS, Paris, France; UNSW Australia, AUSTRALIA

## Abstract

**Objectives:**

HIV-induced immunodeficiency is associated with metabolic abnormalities and systemic inflammation. We investigated the effect of antiretroviral therapy (ART) on restoration of insulin sensitivity, markers of immune activation and inflammation.

**Methods:**

Immunological, metabolic and inflammatory status was assessed at antiretroviral therapy initiation and three years later in 208 patients from the ANRS-COPANA cohort. Patients were compared according to their pre-ART CD4^+^ cell count (group 1: ≤ 200/mm^3^, n = 66 vs. group 2: > 200/mm^3^, n = 142).

**Results:**

Median CD4^+^ cell count increased in both groups after 3 years of successful ART but remained significantly lower in group 1 than in group 2 (404 vs 572 cells/mm^3^). Triglyceride and insulin levels were higher or tended to be higher in group 1 than in group 2 at ART initiation (median: 1.32 vs 0.97 mmol/l, p = 0.04 and 7.6 vs 6.8 IU, p = 0.09, respectively) and remained higher after three years of ART (1.42 vs 1.16 mmol/L, p = 0.0009 and 8.9 vs 7.2 IU, p = 0.01). After adjustment for individual characteristics and antiretroviral therapy regimens (protease inhibitor (PI), zidovudine), insulin levels remained significantly higher in patients with low baseline CD4^+^ cell count. Baseline IL-6, sCD14 and sTNFR2 levels were higher in group 1 than in group 2. Most biomarkers of immune activation/inflammation declined during ART, but IL-6 and hsCRP levels remained higher in patients with low baseline CD4^+^ cell count than in the other patients (median are respectively 1.4 vs 1.1 pg/ml, p = 0.03 and 2.1 vs 1.3 mg/ml, p = 0.07).

**Conclusion:**

After three years of successful ART, low pretreatment CD4^+^ T cell count remained associated with elevated insulin, triglyceride, IL-6 and hsCRP levels. These persistent metabolic and inflammatory abnormalities could contribute to an increased risk of cardiovascular and metabolic disease.

## Introduction

Altered glucose metabolism, including insulin resistance, is more frequent in HIV-infected patients than in the general population and is associated with an excess risk of diabetes [1]. Insulin resistance, generally assessed by increased insulin levels, has been variously attributed to certain antiretroviral drugs (particularly some nucleoside reverse-transcriptase inhibitors (NRTIs) and protease inhibitors (PIs)), antiretroviral therapy (ART)-induced lipodystrophy, and classical risk factors such as age, sex, body mass index and genetic susceptibility [1–9]. In addition, leptin is strongly associated with body fat mass, and adiponectin is a major contributor to insulin sensitivity. Thus the leptin/adiponectin ratio has been shown to be a powerful surrogate marker of insulin resistance in the general population [10]. As well, in HIV-infected patients, we have previously observed that the adiponectin/leptin ratio was associated with insulin sensitivity [11]. Persistent moderate systemic inflammation in HIV-infected patients on ART appears to increase the risk of diabetes [12–14], an association not observed before ART [14, 15]. Markers of systemic inflammation and immune activation are usually elevated in HIV-infected patients, both before and during ART [16–21]. HIV-infected patients show increased microbial translocation, a phenomenon associated with immune activation and inflammation [22] and also with insulin resistance and lipid disorders [23]. Elevated levels of inflammatory markers have also been linked to higher all-cause morbidity, including cardio-vascular diseases, and mortality [24–28].

Low CD4^+^ T cell counts at ART initiation or during follow-up, and a low CD4 nadir, have been linked to an increased risk of new-onset diabetes [2, 3, 29, 30]. We have previously reported an increased risk of insulin resistance among severely immunodeficient ART-naïve patients enrolled in the ANRS CO9 COPANA cohort [15]. The impact of sustained ART on restoration of insulin sensitivity is unknown.

The main objective of this study was to investigate changes in insulin sensitivity and biomarkers of inflammation/immune activation after ART initiation in patients with chronic HIV infection, according to their pretreatment CD4^+^ T cell count. We analyzed the impact of immune restoration and persistent inflammation on insulin sensitivity.

## Methods

### Study design and population

ANRS CO9 COPANA is an ongoing prospective cohort study conducted in 37 hospitals in France. Eight hundred recently diagnosed (<12 months) ART-naive HIV-infected patients were recruited to the cohort between 2004 and 2008. The cohort is funded by Inserm-ANRS and the study was approved by the Paris-Cochin Ethics Committee in July 2003. The research was conducted in accordance with the Declaration of Helsinki; All the participants gave their written informed consent. Socio-demographic, clinical and biological data were collected at enrolment and every 6 months thereafter. Each patient's history of AIDS-defining illnesses, cardiovascular disease, cancer, diabetes or other diseases, and HBV and HCV serostatus were recorded, along with CD4^+^ and CD8^+^ T cell counts and plasma HIV-1 RNA viral load (VL). Fasting total cholesterol, high-density lipoprotein (HDL)- and low-density lipoprotein (LDL)-cholesterol, triglyceride and glucose levels were measured with standard procedures in each center at least once a year.

For this study, we selected the 208 patients who took ART continuously for at least three years and had available frozen samples at ART initiation and three years later. We compared two groups based on the CD4^+^ T cell count at ART initiation: 66 patients with ≤ 200 CD4^+^ cells/mm^3^ (group 1) and 142 patients with > 200 CD4^+^ cells/mm^3^ (group 2).

### Measurements

Clinical and laboratory data were collected before ART initiation and during treatment. Cryopreserved serum and plasma were used for centralized measurements at Tenon Hospital Biochemistry Department (Paris, France). Plasma glucose (hexokinase) and insulin (chemiluminescence immunoassay) were measured on the Architect^®^ Ci8200 analyzer (Abbott). High-sensitivity (hs) CRP was measured by immunonephelometry on an IMMAGE analyzer (Beckman-Coulter). High-sensitivity (hs) IL-6, sCD14, sCD163, sTNFRI and II were measured with enzyme-linked immunosorbent assays (ELISA) (Quantikine^®^, R&D Systems).

Impaired fasting glucose metabolism was defined, in the absence of diabetes, by at least one fasting glycemia value between 5.6 and 6.9 mmol/l. Diabetes was recorded if the patient was receiving antidiabetic treatment or if the fasting glucose was ≥ 7 mmol/l. ART regimens and treatments for comorbidities were recorded every 6 months. To better take into account actual ART drugs exposure during the 3-year study period, we distinguished never-exposed patients, patients who stopped ART drugs for more than 6 months before the 3-year endpoint, and patients still taking ART.

### Statistical analyses

To study the impact of immunodeficiency on insulin sensitivity and inflammatory markers, the patients were categorized according to their CD4^+^ T cell count at ART initiation (≤ 200/mm^3^ versus > 200/mm^3^). Continuous variables were recorded as medians and 25^th^ to 75^th^ percentiles (IQR), and categorical variables as percentages. Non-parametric Wilcoxon tests were used to compare continuous variables, and the χ^2^ or Fisher’s test was used for categorical variables. Pearson correlation coefficients were used to estimate the relation between continuous variables. Comparisons of anthropometric and lipid values between groups 1 and 2 were adjusted for sex in multiple logistic or linear regression models. Multivariate linear regression models were used to examine the influence of the following variables on insulin levels after three years of ART: age and insulin levels at ART initiation, sex, BMI, and ongoing PI or ZDV exposure after three years of ART. Reported p values are those estimated from the Wald test values by SAS GLM. SAS software version 9.3 (SAS institute, Cary, NC, USA) was used for all analyses.

## Results

### Baseline Characteristics

At ART initiation, the median age of the 208 patients was 38 years (IQR 46–32); 30% of the patients were women (n = 63), and 38% originated from sub-Saharan Africa (n = 78). The median CD4^+^ T cell count was 248/mm^3^ (IQR 308–156), and 32% of the patients (n = 66) had a pre-ART count ≤ 200 cells/mm^3^. Median viral load (VL) was 4.8 log_10_ copies/mL (IQR: 5.4–4.3). Eight patients (4%) were coinfected with HCV and 4 (2%) with HBV.

The patients' characteristics are shown in Table 1 according to their CD4^+^ T cell count at ART initiation. Patients in group 1 (pre-ART CD4^+^ T cell count ≤ 200/mm^3^) were slightly older than patients in group 2 (pre-ART CD4^+^ T cell count > 200/mm^3^) (38.5 versus 37.0 years, p = 0.09) and, as expected, had experienced more AIDS-defining events (31.8% versus 3.5%, p<0.0001). There was no difference in gender, geographic origin, HBV and HCV status, and current smoking between the groups. The differences in the proportions of HBV, HCV coinfected patients and current smokers remained not significant between the groups during the study period (data not shown). In the entire study group, only 1 patient reported current drug injection which precludes any comparison.

**Table 1:** Main parameters and their evolution, according to CD4 T-cell count at ART initiation, at three years-ART.

	ART initiation			After 3 years of ART			Comparison of evolution^2^
	CD4+ T-cell counts at ART initiation			CD4+ T-cell counts at ART initiation			
Characteristics	≤ 200 (n = 66) group 1	> 200 (n = 142) group 2	p	≤ 200 (n = 66) group 1	> 200 (n = 142) group 2	p	p
**Baseline Characteristics**							
**Female**, % (n)	33.3 (22)	28.9 (41)	0.51				
**Age at ART initiation**, years	38.5 (33.8; 47.5)	37.0 (30.8; 45.1)	0.09				
**From sub-Saharan Africa**	43.9 (29)	34.5 (49)	0.19				
**Clinical AIDS**, % (n)	31.8 (21)	3.5 (5)	<0.0001				
**Hepatitis C**, % (n)	3.0 (2)	4.2 (6)	0.68				
**Hepatitis B**, % (n)	3.0 (2)	1.4 (2)	0.59				
**Current smoking**, % (n)	25 (16)	29 (41)	0.49				
**Evolution from baseline**							
**CD4+ T-cell counts,** cells/mm^3^	92 (51; 142)	283 (247; 329)	<0.0001	404 (304; 550)	572 (465; 692)	<0.0001	0.08
**CD4/CD8 Ratio**	0.1 (0.1; 0.25)	0.3 (0.2; 0.5)	<0.0001	0.55 (0.33; 0.85)	0.85 (0.63; 1.16)	<0.0001	0.04
**HIV-1 RNA levels**, log_10_ cop/ml	5.2 (4.8; 5.7)	4.7 (4.1; 5.2)	<0.0001				
**HIV-1 RNA levels <50 cop/ml**, % (n)				89.4 (59)	92.3 (131)	0.49	0.77
**Treatment combination**			0.20			0.45	
2 NRTI + 1 PI/r	68.2 (45)	57.0 (81)		53.0 (35)	47.2 (67)		
2 NRTI + 1 NNRTI	22.7 (15)	35.2 (50)		33.3 (22)	42.3 (60)		
Other	9.1 (6)	7.8 (11)		13.6 (9)	10.6 (15)		
**ZDV- containing regimen**, % (n)	42.4 (28)	21.8 (31)	0.002	27.3 (18)	7.0 (10)	<0.0001	
**Total Cholesterol,** mmol/L	4.07 (3.50; 4.70)	4.27 (3.64; 4.88)	0.12 ^1^	5.19 (1.09; 6.01)	5.04 (4.47; 5.67	0.30 ^1^	0.18^1^
**HDL-cholesterol,** mmol/L	0.93 (0.82; 1.18)	1.09 (0.83; 1.29)	0.01 ^1^	1.20 (0.97; 1.63)	1.31 (1.09; 1.64)	0.15 ^1^	0.75^1^
**LDL-cholesterol**, mmol/L	2.40 (1.94; 2.89)	2.66 (2.13; 3.10)	0.06 ^1^	3.20 (2.09; 3.69)	3.06 (2.58; 3.57)	0.54 ^1^	0.09 ^1^
**Triglycerides,** mmol/L	1.32 (0.91; 1.97)	0.97 (0.79; 1.49)	0.04 ^1^	1.42 (1.02; 2.19)	1.16 (0.88; 1.60)	0.0009 ^1^	0.81 ^1^
**BMI**, kg/m^2^	22.8 (20.1; 25.7)	23.3 (21.6; 25.6)	0.27 ^1^	24.7 (22.6; 28.0)	23.7 (21.6; 27.1)	0.12 ^1^	<0.0001^1^
**Impaired fasting glucose (IFG)** ^,^ % (n)	10.9 (7)	14.6 (20)	0.51 ^1^	10.6 (7)	18.3 (26)	0.18 ^1^	0.99 ^1^
**Diabetes,** % (n)	8.4 (6)	6.6 (9)	0.43 ^1^	10.6 (7)	8.5 (12)	0.57 ^1^	0.57 ^1^
**Fasting glucose**, mmol/l	4.9 (4.4; 5.4)	5.1 (4.6; 5.5)	0.08	4.9 (4.3; 5.6)	5.1 (4.7; 5.7)	0.09	0.44
**Fasting insulin**, mUI/L	7.6 (5.2; 17.3)	6.8 (4.9; 9.0)	0.09	8.9 (5.5; 15.1)	7.2 (4.8; 9.5)	0.01	1.00
**Leptin**, ng/mL	4.4 (1.7; 13.1)	4.1 (1.7; 10.2)	0.97 ^1^	7.1 (2.8; 13.7)	5.2 (2.2; 9.6)	0.06 ^1^	0.03
**Adiponectin**, ng/L	4.2 (2.9; 6.9)	4.5 (3.0; 5.7)	0.10 ^1^	4.2 (2.9; 6.2)	4.8 (3.4; 6.8)	0.10 ^1^	0.006
**Leptin/Adiponectin ratio**	1.03 (0.44; 2.54)	1.18 (0.37; 2.54)	0.45 ^1^	1.28 (0.70; 4.1)	1.17 (0.44; 2.46)	0.02 ^1^	0.003

p-value are from rank sum Wilcoxon test or χ^2^/Fisher test; Date are medians and 25^th^ to 75^th^ percentiles or % (frequencies)

NRTI: nucleoside reverse-transcriptase inhibitor; NNRTI: non-nucleoside reverse-transcriptase inhibitor; PI: protease inhibitor

^1^ p-values adjusted for sex

^2^ patients with >200/mm^3^ CD4 T-cell counts (group1) vs. patients with ≤200/mm^3^ CD4 T-cell counts (group2) at ART initiation

### Immunovirological response (Table 1)

At ART initiation the median CD4^+^ T cell count was 92/mm^3^ in group 1 and 283/mm^3^ in group 2 and the CD4^+^/CD8^+^ ratio was lower in group 1 than in group 2 (p<0.0001). After three years of ART, the CD4^+^ T cell count and the CD4^+^/CD8^+^ ratio increased in both groups but remained significantly lower in group 1 than in group 2 (p<0.0001 for both), whereas the proportion of patients with undetectable viral load was similarly high in the two groups (89.4% and 92.3%, respectively).

For most of the 208 patients, first-line ART combined 2 nucleoside reverse-transcriptase inhibitors (NRTI) with either a PI (60.6%) or a non-nucleoside reverse-transcriptase inhibitor (NNRTI) (31.3%). The proportion of patients taking a PI was lower at three years (49.0%), owing to switches to other regimens. Patients with initial CD4^+^ T cell counts ≤ 200/mm^3^, who were more likely to start treatment before 2006, were more often prescribed zidovudine (ZDV) (42.4% versus 21.8%, p = 0.002) and less often tenofovir (TDF) (42.4% versus 59.1%, p = 0.02) than patients with higher initial CD4^+^ cell counts. The proportion of patients whose first-line ART regimen contained abacavir (ABC) was low in both groups (9% in group 1 and 14% in group 2, p = 0.31).

After three years of ART, the proportion of patients still receiving ZDV was lower in both groups than at baseline but remained higher in group 1 than in group 2 (27.3% versus 7.0%, p<0.0001). ZDV was mainly replaced by TDF (48.5% in group 1 versus 70.4% in group 2, p = 0.002) and less often by ABC (16.7% and 19.7%, p = 0.60).

### Metabolic outcomes

Table 1 shows lipid and glucose metabolic status at ART initiation and after three years of continuous ART in the two groups.

BMI did not differ significantly according to the initial CD4^+^ cell count, either before or after three years of ART (p values adjusted for sex = 0.27 and 0.12, respectively). However, patients in group 1 gained significantly more weight during ART than patients in group 2 (p<0.0001).

At ART initiation, total and LDL-cholesterol levels tended to be lower in group 1 than in group 2 (p values adjusted for sex = 0.12 and 0.06, respectively). HDL-cholesterol levels were also lower in group 1 (p value adjusted for sex = 0.01), while triglyceride levels were higher (p value adjusted for sex = 0.04). About 7% of patients in both groups started lipid-lowering therapy during ART. At three years, total, HDL- and LDL-cholesterol levels increased in both groups and no longer differed according to the initial CD4^+^ cell count. Triglyceride levels did not change notably in either group during the three years of follow-up, and thus remained higher in group 1 than in group 2.

The proportion of patients with impaired fasting glucose metabolism or diabetes did not differ between the two groups at ART initiation or at three years, and neither did the median fasting glucose level. The fasting insulin level tended to be higher in group 1 at ART initiation (p = 0.09) and was significantly higher after three years. (8.9 vs 7.2 mU/l, p = 0.01). Fasting glucose values did not change significantly in either group.

Adiponectin levels tended to be lower in group 1 than in group 2, both at ART initiation and at three years (p = 0.10 and 0.10, respectively). Adiponectin levels improved significantly on ART in group 2 but not in group 1. As expected, serum leptin levels varied according to BMI; they increased in both groups, but significantly more markedly in group 1 than in group 2. This led to a significant difference in the leptin/adiponectin ratio after 3 years of ART, which became higher in group 1 than in group 2 (1.28 vs. 1.17, p value = 0.003). Thus, more severe immune deficiency at ART initiation was associated with lower insulin sensitivity after three years of ART.

### Factors associated with diminished insulin sensitivity (Table 2)

**Table 2:** Univariate and multivariate linear regression analysis: role of several parameters on insulin levels after 3 years of ART in HIV-1 infected patients from the ANRS CO9 COPANA Cohort.

	Univariate (n = 194)		Multivariate (n = 194)					
			Model 1		Model 2		Model 3	
Characteristics	β coefficient*	P-value	β coefficient*	P-value	β coefficient*	P-value	β coefficient*	P-value
**CD4+ T-cell counts ≤ 200 /mm** ^**3**^ **at ART initiation (group 1 vs group 2)**	+ 3.32	0.006	+ 2.86	0.02	+ 2.46	0.04	+ 2.71	0.02
**Geographic origin: sub-Saharan Africa** (vs. others)	+ 0.44	0.71	- 0.82	0.53	- 0.69	0.59	- 0.79	0.55
**Female sex** (vs. male)	+ 0.07	0.95	- 0.65	0.62	- 0.56	0.67	- 0.80	0.55
**Age at ART initiation** (for an increase of 1 year)	- 0.04	0.45	- 0.10	0.07	- 0.10	0.06	- 0.10	0.07
**Insulin at ART initiation > 10 mIU/L** (vs. ≤ 10 mIU/L)	+ 4.08	0.002	+ 3.00	0.02	+ 2.41	0.06	+ 2.94	0.02
**BMI at 3 years of ART** (for an increase of 1 kg/m^2^)	+ 0.40	0.003	+ 0.39	0.005	+ 0.47	0.001	+ 0.39	0.006
**ZDV exposure at 3 years**								
0 or < 1 month	ref				ref			
Interrupted	- 1.25	0.41			- 2.17	0.15		
Still exposed	+ 3.50	0.03			+ 2.57	0.10		
**PI exposure**								
0 or < 1 month	ref						ref	
Interrupted	+ 0.60	0.77					+ 0.24	0.89
Still exposed	+ 2.20	0.10					+ 1.50	0.24

* From linear regression model: the model provides the difference in mean insulin levels between the modalities of a variable

We then used univariate and multivariate linear variation analyses to study the role of selected factors in the diminished insulin sensitivity, based on insulin levels after three years of ART (Table 2). These analyses involved the 194 patients for whom complete data were available. In univariate analysis, patients with low CD4^+^ cell counts (≤ versus > 200), an insulin level above 10 mIU/L at ART initiation, or a large increase in BMI (as a continuous variable) after three years of ART were more likely to have elevated insulin levels after three years of ART (p = 0.006, 0.002 and 0.003, respectively). Patients still exposed to ZDV at three years had higher insulin levels than patients never/no longer exposed to ZDV (p = 0.003), while those still exposed to a PI at three years of ART tended, but not significantly, to have higher insulin levels than patients never/no longer exposed to a PI (p = 0.10).

After adjustment for geographic origin, sex, age at ART initiation, BMI after three years of ART and ongoing exposure to ZDV (model 2) or PIs (model 3), insulin levels after three years of ART remained higher in group 1 than in group 2 (p = 0.04 and 0.02, respectively), indicating that an initial CD4^+^ cell count ≤ 200/mm^3^ is a risk factor for an altered insulin sensitivity after 3 years of successful ART. Similar results were obtained when only patients with VL < 50 copies/mL at three years of ART were taken into account.

### Markers of inflammation and immune activation (Fig 1)

**Fig 1:**
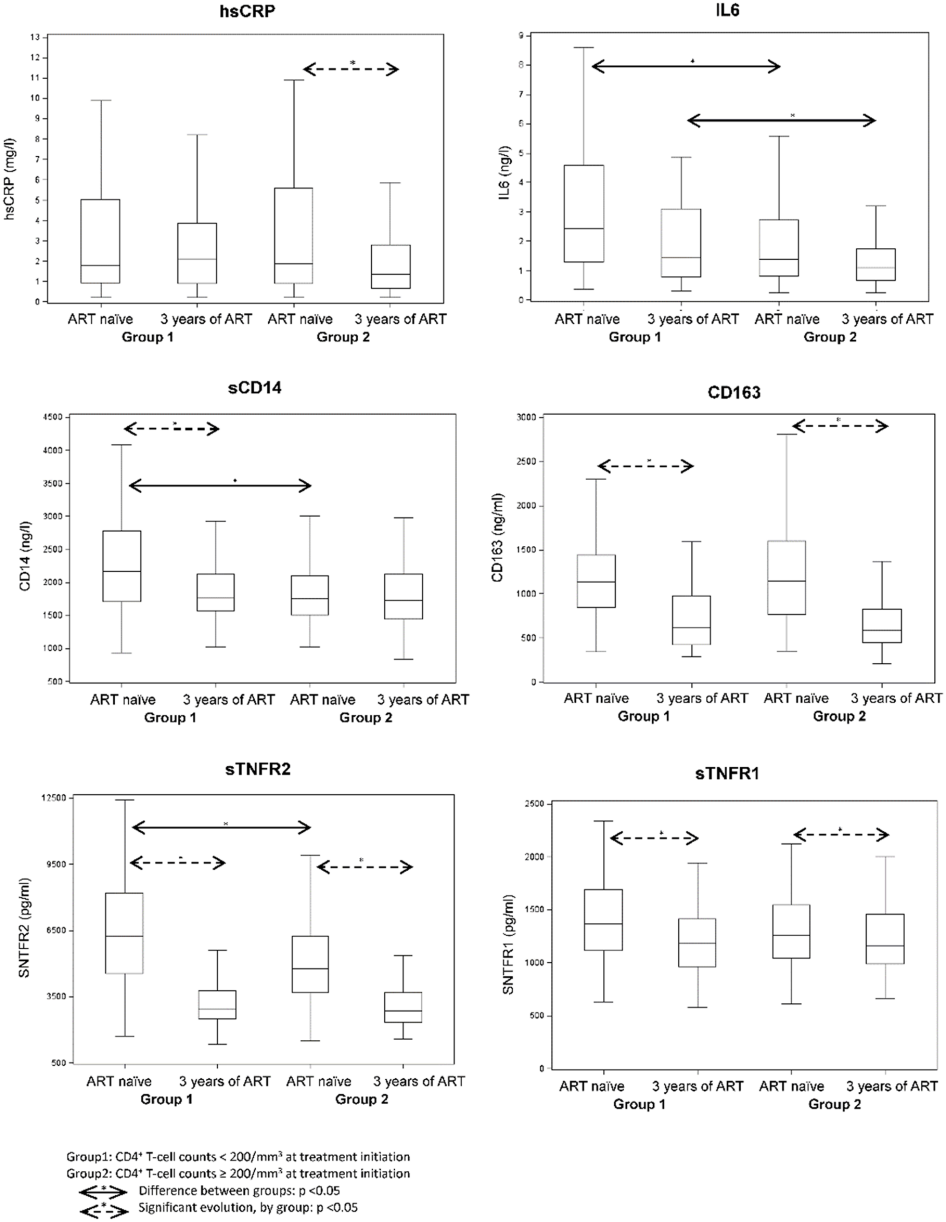
Distribution of inflammatory markers according to CD4 T cell count at ART initiation.

At ART initiation, levels of IL-6 and sTNFRII were higher in group 1 than in group 2 (p = 0.0009 and <0.0001, respectively), whereas hsCRP levels did not differ. During the three years of ART, the levels of these markers evolved differently. IL-6 levels tended to decline in both groups but remained higher in group 1 than in group 2 (p = 0.03). sTNFRI and sTNFRII fell significantly, to similar levels in the two groups. Levels of hsCRP were unmodified in group 1 but fell significantly in group 2 (p = 0.03). Thus, after 3 years of ART, the hsCRP level tended to be higher in group 1 than in group 2 (p = 0.07).

In group 1, sCD14 levels, which were higher than in group 2 at ART initiation, fell to values similar to those observed in group 2 at ART initiation and after three years of ART. sCD163 levels were high at ART initiation in both groups and declined to similar levels after 3 years of ART (p = 0.55). Of note, higher insulin levels at 3 years of ART were associated with higher hsCRP and sCD163 levels, even in patients with undetectable VL, while other parameters were not linked to insulin levels (data not shown).

## Discussion

Several cross-sectional studies have shown a relationship between insulin sensitivity and the CD4^+^ cell count, both in ART-naïve patients and in patients on ART [14, 31, 35, 36]. However, to our knowledge, this is the first study to simultaneously evaluate changes in insulin sensitivity and markers of immune activation and inflammation during long-term ART.

We examined changes in insulin sensitivity and markers of inflammation and immune activation in HIV-infected patients according to their CD4^+^ cell count at ART initiation (≤ or > 200 cells/mm^3^). After three years of successful ART, patients who had been profoundly immunodeficient owing to late ART initiation had lower CD4^+^ cell counts and CD4/CD8 ratios and, importantly, lower insulin sensitivity and higher circulating IL-6 levels (with a trend towards higher hsCRP levels) than patients in the same cohort who were less severely immunodeficient at ART initiation.

At 3 years, total, LDL- and HDL-cholesterol levels no longer differed between patients with baseline CD4^+^ cell counts below and above 200/mm3, related to virologic suppression and immune restoration. In contrast, triglyceride levels remained higher in the patients who were more immunodeficient at ART initiation, even though similar proportions of patients in the two groups received PI-based ART. These results are in keeping with those of previous studies showing that HIV infection alters the lipid profile, notably with low cholesterol and high triglyceride levels [31–32], and that these lipid alterations are linked to immune deficiency and/or HIV replication, independently of cachexia and major weight loss [15].

We previously showed that insulin sensitivity was more strongly impaired in severely immunodeficient ART-naïve patients than in their less immunodeficient counterparts [15]; we observed the same trend in the present group which originates from the same cohort. Furthermore, other studies showed similar results [31, 33]. Importantly, after three years of successful ART, insulin levels were higher in patients who started treatment at low CD4^+^ cell counts (≤ 200/mm^3^). This difference persisted after taking into account other factors that might influence glucose metabolism, such as gender, geographic origin, the initial insulin level, and BMI and even after taking into account the use of PIs or ZDV, which are known to increase the risk of insulin resistance and diabetes [4, 6, 12, 34]. Thus, three years of effective ART had little impact on insulin resistance associated with pretreatment immunodeficiency.

Markers of inflammation and immune activation improved during ART. Interestingly, after 3 years of successful ART, hsCRP and hsIL-6 levels were similar to those we recently observed in another cohort of patients on long-term effective ART (APROCO-COPILOTE), which were relatively low but higher than in uninfected subjects [21]. As expected, markers of inflammation and immune activation were more markedly elevated in the patients who were most immunocompromised before starting ART. After three years of ART, even if CD4^+^ cell counts remained lower in patients with lower pretreatment counts, the level of these markers no longer differed between the groups, arguing for a major role of the viral suppression that drives immune restoration. Nevertheless, hsIL-6 and, marginally, hsCRP levels remained elevated, indicating persistent low-grade systemic inflammation in patients with lower CD4 counts at ART initiation.

Few studies have focused on markers of inflammation and immune activation according to the CD4^+^ cell count or HIV viral load. In the Veterans Aging Cohort Study (VACS) [37], IL-6 and sCD14 levels were higher in ART-naïve patients with CD4^+^ cell counts < 200/mm^3^, and this remained true for ART-treated patients whose CD4^+^ cell count remained below 200/mm^3^. We have also previously observed that TNF-α, sTNFRI, sTNFRII and IL-6 levels are inversely related to the CD4^+^ cell count in ART-naïve patients [15]. The level of IL-6 was previously shown to be related to the CD4 nadir and to high levels viral load [20]. It should be noted, however, that these studies were cross-sectional and did not therefore analyze changes after ART initiation.

Changes in markers of inflammation and immune activation during ART have been widely studied. IL-6 levels fell in two studies of patients with initial median CD4^+^ cell counts of respectively 240/mm^3^ [38] and 315/mm^3^ [39], while they did not change in another study in which the initial median CD4^+^ cell count was higher (431/mm^3^) [40]. sTNFRI and sTNFRII levels fell after 96 weeks of ART [38], whereas CRP levels did not change [38–40]. ART was associated with an improvement in the activation markers sCD14 and sCD163 [41–42]. However, these studies did not interpret changes in marker levels according to the initial CD4+ cell count. Our results, showing similarly elevated sCD14 levels after three years of successful ART in patients with pre-ART CD4 cell counts above and below 200/mm^3^, are in keeping with reports that control of HIV replication does not totally suppress gut barrier alterations and microbial translocation, which are important drivers of persistent immune activation [22]. Chronic monocyte activation, associated with persistent low-grade systemic inflammation in patients with lower CD4 counts at ART initiation, probably plays a role in their lower insulin sensitivity, as suggested by the association between insulin levels at 3 years of ART and higher hsCRP and sCD163 levels. This is in accordance with previous studies showing that insulin resistance is associated with immune activation in the general population [43].

Compared with historic studies using first-generation PIs such as indinavir, insulin resistance was moderate and the incidence of diabetes was low in our cohort, in keeping with the use of more recent ART regimens. In addition, levels of inflammatory markers after 3 years of effective ART were not very high, except for sCD14, which remained elevated regardless of the pre-ART CD4 cell count. One limitation of our study is that the two groups of patients were not included during the same calendar periods, due to the evolving recommendations in the CD4 criteria of ART initiation, and thus that the ART combinations were different. We have carefully adjusted for these ART differences in our study and show that the differences in insulin sensitivity persisted.

In conclusion, we found that markers of metabolic abnormalities and inflammation remained elevated, albeit moderately, after 3 years of successful ART in patients who started ART at low CD4 cell counts. These persistent abnormalities are known to be associated with an increased long-term risk of cardiovascular and metabolic disease. It remains to be seen whether these patients ultimately recover marker values similar to those observed in patients who begin ART at higher CD4 cell counts.
